# Counterfactual analysis of the impact of the first two waves of the COVID-19 pandemic on the reporting and registration of missing people in India

**DOI:** 10.1057/s41599-022-01426-8

**Published:** 2022-11-12

**Authors:** Kandaswamy Paramasivan, Brinda Subramani, Nandan Sudarsanam

**Affiliations:** 1 Directorate of Vigilance and Anti-corruption, Government of Tamil Nadu, Chennai, India; 2 Tamil Nadu Police Department, Government of Tamil Nadu, Chennai, India; 3 Department of Management Studies, Indian Institute of Technology, Madras, Chennai, India; 4 Robert Bosch Centre for Data Science and AI, Indian Institute of Technology, Madras, Chennai, India

**Keywords:** Criminology, Sociology

## Abstract

The primary duty of law enforcement agencies is to ensure that a victim has the necessary information and access to the relevant tools required to seek justice. In India, complex cases such as bodily offences and property crimes capture the work and efforts of many agencies involved; however, cases related to missing persons are not often accorded similar priority or seriousness. The COVID-19 pandemic and subsequent lockdowns have added further challenges to this scenario. The government-mandated lockdowns in Tamil Nadu generally exacerbated difficult socio-economic and living conditions, thereby directly or indirectly contributing to an increased load of missing person cases. This study aims to assess and identify the impact of mobility on reporting and registration of missing persons. By adopting an auto-regressive neural networks method, this study uses a counterfactual analysis of registered missing person cases during the government-mandated lockdowns in response to the global pandemic in 2020 and 2021. The registered cases are calculated based on the daily count of cases for eleven years in Tamil Nadu, India. The lockdowns identify eight different time windows to determine the impact of mobility on the registration of cases. While there has been no significant or drastic change over the pre-pandemic period, during the pandemic, especially during the restrictive phases of the pandemic, there was a sharp fall in cases compared to the counterfactual predicted (effect sizes: −0.981 and −0.74 in 2020 and 2021), signalling towards a choked mechanism of reporting. In contrast, when most mobility restrictions were removed, an increase in cases (effect sizes of +0.931 and 0.834 in 2020 and 2021) pointed to restored and enabled reporting channels. The research findings emphasise the significance of mobility as a factor in influencing the reporting and registration of missing persons and the need to ensure this continues to help families find redress.

## Introduction

### Understanding the problem of missing persons during the pre-pandemic period

Crime is ubiquitous across most societies and nation-states. The plight of victims, for whose remedy the criminal justice system has been institutionalised, is sometimes overlooked in the system. However, police act as the first responders in most instances to work towards restoring normalcy. This is nevertheless determined by situational factors such as the victims’ ability, accessibility, and earnestness to reach out to the agencies for relief and redressal.

While cases related to missing persons are not considered crimes by law, the causes leading to missing persons and the consequences thereafter could potentially be criminal. Moreover, the agony that the kith and kin of missing persons undergo demands that justice be served. Missing persons cases are often a protracted battle, unlike violent cases of crime such as murder and other heinous crimes such as rape and dacoity, that are solved reasonably promptly. The missing person’s family and friends live in hope of the return of their loved ones, their grief and vulnerability intensifying with each passing day as they continue to remain unfound. Moreover, several law enforcement agencies suggest that the longer it takes to find the person, the lower the chances are of finding him or her. The impact on the family and the psychological distress that they experience present a challenge to the police system.

**Fig. 1:**
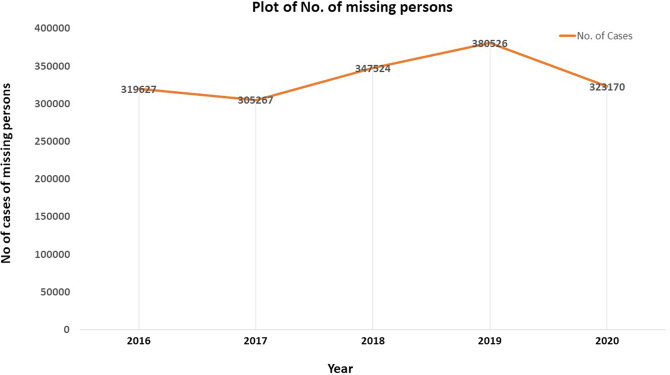
Plot of the number of missing person cases in India during the years 2016–2020. *x*-axis: year. *y*-axis: number of cases of missing persons. Source: National Crime Records Bureau, New Delhi.

The cases of missing persons are unique in several ways when compared to other criminal cases, not only in incidence but also in reporting such instances for tracing and locating the missing person. These cases are a bigger problem and challenge to law enforcement, and they deserve greater attention and a more effective response from organisations and governments. There is emotional grief for the kith and kin of the missing person, particularly if the missing person happens to be the breadwinner of the family (Testoni et al., 2020). It is widely acknowledged that the police have adopted many innovative methods worldwide to locate missing persons (Ferguson and Soave, 2020; Pier et al., 2021). For example, UK police have begun assessing risk factors for missing persons, assisting in the implementation of appropriate operational activities. Additionally, they have been using publicity methods to invoke community participation in finding the missing person. (Greene, 2020). One may think that, with the advancement of technology, the penetration of social media, and greater awareness among individuals, the number of missing persons reported would dwindle, and the number of missing persons traced by law enforcement or others would increase. However, data from 2017, 2018, and 2019 points to the contrary, indicating an increase in reported cases of missing persons in India. (See Fig. 1).

Additionally, research points out that “there is an exceedingly complex web of behaviours and responses surrounding the phenomenon of missing persons.” (Nicholas et al., 2015). Locating a missing person is one of the most challenging tasks for the police force due to the number of resources required, the psychological distress of the family, and the often limited information available. The search process for a missing person, whether alive or dead, is an overarching concept with an integrated, multidisciplinary, and multi-agency approach seeking committed cooperation by all the stakeholders involved (Salado Puerto et al., 2021). Further, tracing and locating missing persons is more difficult for police in terms of effort, resources, time, and strain involved as compared to investigating property crimes or violent crimes (Shalev and Pakes, 2014).

The aforementioned discussion is a post-mortem analysis of what was done in the aftermath of an individual found missing. The present paper is primarily concerned with the factors that prevent complainants from reporting cases of missing persons to the police. In order to understand the challenges related to the reporting of missing persons, there needs to be an understanding of the conditions under which individuals go missing in the first place. The following section provides details of the causes behind missing persons, both in India and in other countries, during regular situations or pre-pandemic times as well as pandemic times, to help appreciate the results of this study.

### Major reasons for missing persons in India during the pre-pandemic period

A deeper look at cases related to missing persons provides a broad categorisation of reasons for individuals to go missing. Analysis of the data consisting of the First Information Report (basic crime record for investigation) and other information available with the district crime records bureau in Tamil Nadu suggests that individuals go missing for various reasons that are often related to socio-economic factors as well as cultural factors. The cases can be broadly categorised as follows: (a) unintentional absence: the missing people who had become disoriented or drifted; as well as those who have been forced to be absent, such as children or the elderly who were separated from their guardians in fairs or large gatherings such as festivals or religious conferences, and were unable to communicate with them; or those who have been kidnapped; (b) intentional absence: those who have voluntarily left for a variety of reasons, such as elopement or academic failure in order to escape. Those who voluntarily go missing do not wish to be found, thus making these cases much more difficult to resolve. The number of missing cases due to elopement is rampant given the continued practice of forced arranged marriages, where factors beyond the mutual liking of the partners play a role. Elopement, at times, is the only way to save the lives of individuals who otherwise stand a very high chance of being killed on account of the inter-caste or inter-religion nuptial knot they get involved in. Also in recent times, academic failure as a reason behind missing cases is fast rising in India as peer pressure and societal expectations are paramount over an individuals’ calibre, likes, and dislikes.

When discussing intentional absence, examples such as individuals escaping abuse from a care home or an assisted living facility, come to mind. These individuals want to disappear from their places of residence on their own volition. This is a common category of missing persons in India; a significant chunk are those who willingly leave their regular place of residence. Bankruptcy and suicide are other examples and forms of intentional absence. (Laura van, 2013). These individuals have control and clarity over their actions, they choose and plan the design that suits their needs. Disorientation caused by substance abuse also falls under this category.

Certain scenarios of missing persons are also considered an involuntary phenomenon. These include missing persons cases influenced by of a third person or party; immoral trafficking, possible abduction or kidnapping. This category primarily includes those who are missing against their will; such as the cases of women missing, which suggests yet another dimension—that of continuing gender-based violence in society. (Ambler, 2014). Amartya Sen, for instance, in one of his works, mentioned that the skewed sex ratio in the country can be attributed to gender discrimination and disadvantage. (Sen, 1990). This category is often absent from society’s sociological imaginations, let alone law enforcement agencies. The last scenario includes individuals lost due to injury or mental illness such as Parkinson’s or unfortunate accidents (Greene, 2020). Whatever the reason might be, in the pre-pandemic times, law enforcement agencies were intimated, and a suitable response was taken in the form of registration of the case at the least.

### Missing persons in other parts of the world during the pre-pandemic period

Many individuals go missing in the war-torn areas of Western Asian countries such as Syria, Iran, and Iraq; the civil strife areas of Africa, the border areas of Mexico-US or North and South Korea mainly because of the illegal migrants’ issues. In a similar situation, the Uighur Muslims and Tibetan Buddhists in China and the political dissidents in Russia are prone to criminal detentions and exterminations. However, the cases in India are relatively crime-free. That does not mean that there is no crime associated with the missing people. Nevertheless, before delving into the Indian scenario, a brief mention of the circumstances in each of the aforementioned instances is made in the following section.

Countries facing constant turmoil like Syria, Afghanistan, Iran, and Iraq witness multiple situations of missing persons. For instance, the U.N. estimates that over 100,000 individuals are missing from Syria, primarily due to “summary executions, arbitrary and incommunicado detention, kidnapping and abduction, as well as combatants and civilians missing as a direct result of fighting and the day-to-day ravages of war and war crimes.” (ICMP Syria, 2021). These apply to other war-torn and civil war-affected areas as well. It is important to note that the missing person phenomenon is also rampant in developed countries like the USA (FBI, 2019) and Europe (AMBER Alert Europe, 2022).

These reports suggest that around 600,000 individuals go missing in each region. Moreover, in autocratic countries like China, there are unique cases of disappearances related to political dissidents and/or the extermination of individuals and cultures that are not seen as being oriented towards the existing government. A case in point is Uighur Muslims (APNews, 2020).

### Cases of missing persons during the pandemic period

Numerous studies have been conducted to understand the impact of the COVID-19 pandemic on various aspects of our lives. However, there seems to be a lack of data regarding the impact of the pandemic on the reporting and tracking of missing persons (Calderon-Anyosa et al., 2021). Another aspect that has been understudied relates to the challenges faced by law enforcement agencies in dealing with cases related to missing persons. The absence of witnesses and public transport are reasons individuals are restrained from asking for help, which is a significant factor in such instances (Marks, 2021). The United Kingdom’s National Crime Agency’s Missing Persons Unit report suggests relationship issues and mental worries as primary reasons for individuals missing during the pandemic. The report also suggested increasing domestic violence against women and children, which led to them going missing (Krubiner et al., 2021). The various lockdowns in Tamil Nadu might have resulted in safer conditions for children in general as the chances of being exposed to potential harm such as abduction and human trafficking were very low. On the other hand, adults continued to be at high risk given the COVID-induced forced migrations and loss of livelihoods and depressing economic affairs, thereby making them more vulnerable (Freya O’Brien et al., 2021). The reasons for missing individuals are plenty. However, the present study aims to study how an unprecedented pandemic as well as factors independent of a pandemic situation can affect the reporting of a case.

### The role of situational factors in the reporting and registration of missing persons cases during the pandemic period

The lockdowns, which resulted in restricted mobility, mass exodus of migrants, and a general maintenance of curfew measures, increased demands on law enforcement agencies. Law enforcement authorities remained overstretched and therefore unable to effectively support victims of crime, which made the reporting of crime more challenging overall. The severe restrictions on vehicles, both public and private, comprised a vital hurdle for complainants wanting to file complaints at the police station. In addition, the general fear of contracting COVID-19 also posed a challenge. Such exceptional circumstances during the pandemic brought considerable challenges to the complainants’ ability to reach relief centres. This was also a time which critically affected the overstretched first responders, whose resources were largely redeployed to address the pandemic. Adjusting for mobility during the pandemic-induced lockdowns illustrates mixed results of increasing and decreasing cases related to missing persons when compared with the counterfactuals—primarily due to the operational efficiency and effectiveness of the reporting mechanism during normal periods. The escalation of registered cases could be attributed to the improved channels of reporting missing persons cases during the relevant period. In contrast, a decline in recorded case levels indicates a clogged reporting medium, including the shared commitment of police, non-profit organisations, and other agencies involved in work relating to exceptional circumstances. The empirical study setup, which is made up of eight time windows with different levels of pandemic severity and restrictions, gives us a chance to look at the recorded levels in the context of enabling and impeding crime reporting mechanisms after mobility has been taken into account.

### Motivation for the research

Descriptive statistical tools and conventional forecasting methods such as auto-regressive integrated moving average (ARIMA), Holt-winters, and exponential smoothing have been very popular with practitioners and researchers in criminal justice administration, police science, criminology, and related fields. Though these methods have been in vogue for a few decades, there are certain shortcomings, such as the models’ instability when the data is volatile and has rapid fluctuations. Further, there is a need for manual intervention by the modeller to specify the functional form. With the advent of massive advancements in computing power, besides easy accessibility and availability, the landscape of time-series forecasting has changed to newer arenas of deep learning, such as the usage of recurrent neural networks from the early domain of conventional statistical forecasting techniques.

The most important advantages of an auto-regressive neural network (ARNN) are its accuracy in prediction, its capability to work with sparse data with missing values, and its ability to predict even with little past data as it learns from other similar time series.

Furthermore, very few studies cover the longer span of two pandemic waves, giving it a longitudinal approach. Notably, there are no research articles based on field-level actual primary data in peer-reviewed journals studying the effect of the pandemic waves on the reporting of crimes in general and missing persons in particular in the Indian context. Research findings borne out of studies on other high-income countries may not necessarily apply to India’s social-political-economic and cultural milieu. Hence, an investigation of data specific to India would be relevant to addressing current challenges and informing future policies.

The pandemic and lockdown greatly impacted the cases of missing persons in India. Given the extent of circular migration and migrant labour as well as the geographical vastness of the land, the pandemic aggravated the case for this group of individuals.(Khanna, 2020) Many guest workers (migrant labourers) in India who tried to return to their domicile state were found missing but were not reported (Jesline et al., 2021; Irudaya Rajan et al., 2020). Interestingly, a discussion of missing persons case shapes up as a case of double jeopardy. Unlike other criminal cases, the victim is unheard of and underrepresented in this case. This becomes a more complex issue when those trying to file a complaint are further restricted by an external restriction to report, resulting in primary and secondary instances of victimisation of the missing individual during the pandemic-induced lockdowns. It is imperative to acknowledge the pivotal role of the police in India in this context, more so because police are the first responders to missing individuals. Unlike in other nations, where Interpol databases or non-state organisations aid in such cases, the police in India assumes a singularly vital role.

In supposedly normal circumstances, one cannot study the factors that would have otherwise determined crime reporting and FIR registration, thereby setting the law into motion. The pandemic and the lockdowns provide a natural experiment setting to understand the latent factors that enable crime registration, which are not easily decipherable in normal times. However, the unprecedented pandemic-induced changes in mobility and a host of other exceptional conditions provided an opportunity for a natural experiment in the registration of missing persons’ cases, particularly in the context of the inhibiting mechanism of reporting such cases, in varying mobility trends over eight time windows covering both waves of the pandemic.

## Methods

### Material—natural experiment

The lockdowns brought on by the COVID-19 pandemic significantly impacted mobility. In this context, this paper explores the “registration of crime” and analyses the influence of various mobility trends on the agents that facilitate and obstruct the reporting of crime. The study focused on the state of Tamil Nadu, which is the sixth most populous state in India with an estimated 80 million residents and a land area of about 130,000 square kilometres. The police station is the central point of contact for crime victims, who can come in and file complaints and grievances, primarily using the First Information Report (FIR). Section 154 of the Indian Criminal Procedure Code gives FIRs legal standing, and it is the station house officer’s duty to document any information the complainant provides to the police in writing in order to register all cognisable offences. Although Section 154 claims that it is only for cognizable offences and, as previously stated, missing persons cases are not crimes by themselves, a FIR is still filed in cases involving missing persons in the same way as in other crimes. In 2019, 1356 police stations across 200 police subdivisions, 38 police districts, and 9 commissionerates handled a total of 168116 FIRs related to all IPC-related offences. 16238 cases of missing persons were reported, representing 9.65 percent of all crimes, making it a significant category (NCRB 2020).

**Fig. 2:**
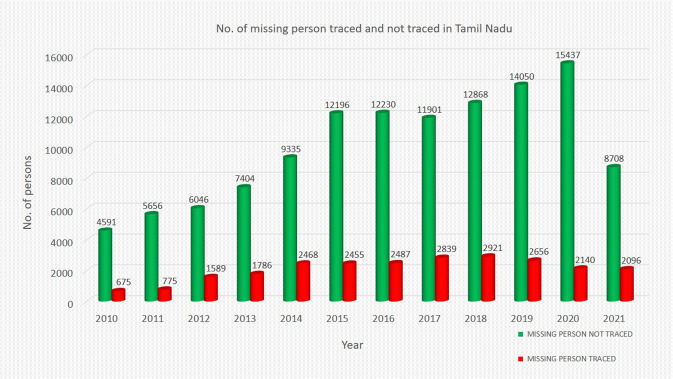
Number of missing person cases (traced and not traced) in Tamil Nadu during the decade 2010–2021. *x*-axis: year. *y*-axis: number of missing person cases in Tamil Nadu.

**Fig. 3:**
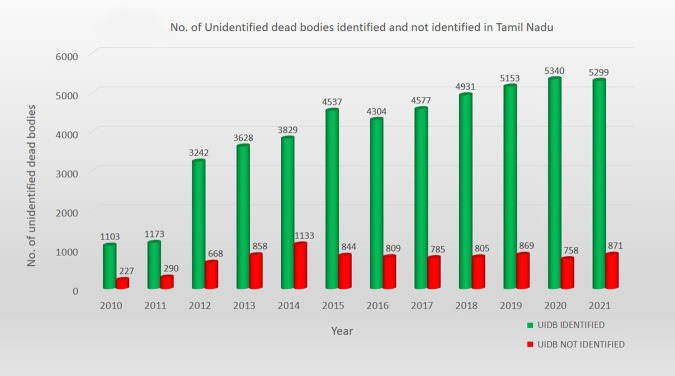
Number of unidentified dead body (UIDB) cases (traced and not traced) in Tamil Nadu during the decade 2010–2021. *x*-axis: year. *y*-axis: number of UIDB cases in Tamil Nadu.

The growing number of missing persons (traced and untraced) during the last decade is perhaps attributed to improved channels of reporting missing persons. A very similar trend to the registered cases of unidentified dead bodies is also seen (see Figs. 2 and 3). The crucial initiatives that paved the way for such a substantial upward climb in the decade ending in 2015 include the opening of the option of online filing of complaints related to missing persons and the sharing of information in the public domain.

The Crime and Criminal Tracking Network and Systems (CCTNS), a police website that stores all crime data, was adopted nationwide. In Tamil Nadu, a host of services were made available to the public, and one of the most important ones that were launched in 2015 pertains to direct access to databases for the public. The complainant could directly use the search option with some features of the person, such as gender, age, height range, complexion, other visible identification marks, and colour of the dress of the person when last seen, to look for the missing person.

**Fig. 4:**
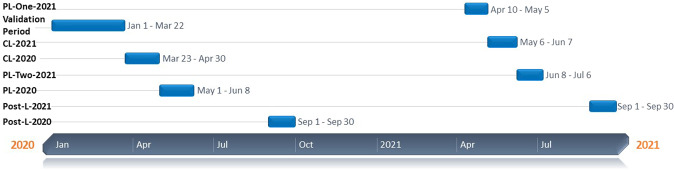
Timeline of the first two waves of COVID-19 lockdowns in Tamil Nadu, India. Horizontal axis: discrete intervals—various lockdown phases in both waves in Tamil Nadu. Vertical axis: time period of various lockdown.

The present study focuses on the pandemic times to understand how the number of FIRs, in the cases of missing persons, is affected during the different phases of lockdown and the preceding and subsequent periods, given the constraints on mobility. In this context, the periods are divided into the following categories: Complete Lockdown (CL) and Partial Lockdown (PL), based on the movement restrictions imposed by the government. These restrictive orders were in place during both the pandemic waves in 2020 and 2021 but differed in length. While the partial lockdown witnessed partial and periodic relaxations in mobility and allowed specific services, goods, individuals, and business establishments to operate, complete lockdowns were severe in the restrictions imposed (see Fig. 4). Figure 4 clearly illustrates the phases of CLs and PLs. It is essential to note this categorisation as it plays a crucial role in this study, through ARNN, of how the mobility restrictions and relaxations impacted the registration of cases.

### Wave one

The Government of India, and thereby the Government of Tamil Nadu, imposed Complete Lockdowns for a total of 38 days between March 23, 2020 and April 30, 2020. During partial lockdown in the period between May 1, 2020 and June 8, 2020, for 38 days, which equals the same number of days as CL, certain restrictions were lifted on the movement of people and vehicles. The post-lockdown phase can be considered September 1, 2020 onwards, when most of the mobility curbs were lifted.

### Wave two

As the COVID-19 cases saw an upsurge, so did the return of the lockdown phase. During the second wave, with knowledge of hindsight, the government initially launched a mildly restrictive order with Partial Lockdown (one) between April 10, 2021 and May 5, 2021. However, with a massive increase in caseload and the ensuing need to curtail the spread of the virus, a complete lockdown with strict stay-at-home orders was enforced between May 6, 2021 and June 7, 2021, which was followed by a period of relaxation in mobility, in the form of Partial Lockdown (two), which was implemented between June 8, 2021 and July 6, 2021. Hence, the complete lockdown was sandwiched between the two partial lockdowns on either side of the timeline. Finally, between September 1, 2021 and September 30, 2021, there was a post-lockdown phase when almost all the curbs were lifted off.

### Method for time-series forecast

This study employs the DeepAR forecasting algorithm, a recurrent neural network (RNN) based model, to forecast the daily count of missing persons and unidentified dead bodies. It consists of an RNN architecture that is ideal for modelling sequential data and a likelihood function that is used to generate a probabilistic forecast (Salinas et al., 2020). This model presents four main advantages over the other models used to forecast crime. First, ARNN makes it possible to model complex time-series processes with no assumptions about the data or any assistance from the modeller in choosing the parameters for the model. This is unlike conventional models, such as the ARIMA, which need the order of the process to be specified. Second, the probabilistic forecast provided by the likelihood function improves the reliability and confidence in its predictions. Third, deepAR models can employ likelihood functions specific to positive count data (crime data falls under this category), like negative binomial distribution, which augments accuracy. Fourth, conventional time-series forecasting techniques like auto-regressive integrated moving average (ARIMA) and exponential smoothing have been used consistently to get stable forecasts for different time-series data. However, these methods are unsuitable when rapid changes, moving holidays, and seasonality factors are in the data. Finally, the model can identify the significant trend and seasonal components and create new features, making it superior in forecasting to conventional statistical models. This model is implemented in Python using the gluonTS package (Alexandrov et al., 2019).

In model building in machine learning and deep learning, three data sets are involved: training, validation, and actual forecast (test). Usually, the training data is large, where the model’s parameters are estimated. The estimation of the appropriate parameters is done in such a manner that the difference between the actual and predicted is the least. These parameters are used in the validation phase, where unseen data is shown to the model for prediction. The error is calculated to check prediction accuracy using an appropriate error metric. Some modellers do the tuning of the parameters to enhance the accuracy and avoid overfitting the model with the data. Finally, the model developed after validation is used for the prediction data.

#### Training phase

**Fig. 5:**
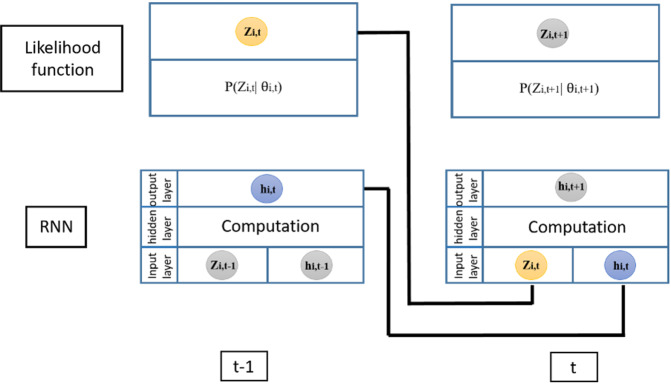
Schematic diagram of DeepAR architecture.

Consider a time series *i* with a total of *t*0 time steps, where *t*0 represents the last observation for the time series *i* or the training range, and *T* represents the last time step till which forecasting is done, or the prediction range. The input to the RNN at time step *t* is the value of the time series and the output from the RNN in the previous time step *t* *–* *1*, are represented by *zi, t* − *1* and *hi, t* − *1*, respectively. These inputs, along with the parameters of the RNN, can be used to produce the output of the RNN at the current time step *t*. Subsequently, this output from the RNN and its parameters are used to parametrise the negative binomial likelihood function (see Fig. 5).

#### Validation phase

During the validation phase, the model is used to forecast from the time step next to the end of training data. The quality of these forecasts is evaluated using an appropriate error metric. This validation of the models’ forecast is possible as the observed values of the time series during the validation phase are known. The forecasts from Auto-regressive Recurrent Neural Network are compared with the predictions from different state-of-the-art forecasting models, such as Auto-regressive Integrated Moving Average (ARIMA), Generalised Additive Model (GAM), Bayesian Structural Time Series (BSTS), and Holt-Winters, and were found to be the most accurate, recording the least error. Weighted Mean Absolute Percentage Error (WMAPE) is used as the performance metric as it is unit-free and can be interpreted easily. Furthermore, WMAPE is more appropriate than the commonly used measure MAPE because the number of missing person cases on certain days may be zero, resulting in an infinite value for MAPE, whereas WMAPE remains finite.

#### Prediction phase

Once the model parameters are estimated, a sample is drawn from the likelihood function for time steps *t* > *t*_o_. This represents the prediction at that time step, which is in turn used to calculate the prediction of the neural network at the next time step. This process is repeated for *n* times (specified by the modeller), producing *n* different traces, each representing a forecast from time step: *T*. These *n* traces of forecast are used to calculate the confidence interval for the predictions.

In this study, a 95% confidence interval is constructed using 0.025 and 0.975 quantiles that were calculated using the values of *n* traces at each time step *t*. Also, as the value of *n* increases, the median of the forecast becomes more reliable.

#### Quantifying the effect of lockdown

The counterfactual forecasted daily count of registered cases of missing persons is the predicted value, with the assumption being that there will be no lockdowns in 2020 and 2021. The model provides the confidence interval for the counterfactual prediction of the response time series, which, when subtracted from the actual, provides the confidence interval estimate of the causal impact on the response variable. So, the model shows both the predicted (the counterfactual) and actual (or daily count) distributions of crime.

Using the Shapiro–Wilk test, we verify the normality assumption for the distribution of the counterfactual time series and the actual registered cases of missing persons. The null hypothesis of this test is that the population is normally distributed. As a result, if the *p*-value is less than the selected alpha level, the null hypothesis is rejected, and evidence that the data being tested is not normally distributed is provided. A *t*-test is used to find out if the difference was statistically significant if the normality requirements are met.

However, a Wilcoxon sign-rank test is used in place of a *t*-test for non-normal distribution. The Wilcoxon test determines whether there is a significant difference between the means of two groups. Since the Wilcoxon test is non-parametric, it is subject to far fewer assumptions than tests that are parametric, like *t-*tests.

We use Cliff’s Delta measure to figure out the size of these effects instead of just assuming that the pandemic had an effect on registered missing person cases. This is because Cliff’s Delta measure is more reliable if the parametric assumptions are not met (Li, 2016).

### Method of measurement of mobility

One of the immediate and direct consequences of movement restrictions was its effect on the mobility of people and vehicles, which was measured using Google’s COVID-19 community mobility reports (CMR).The CMR gathers data from both handheld devices and public domain, particularly from those allowing recording of location history, thereby importantly giving two sets of information—a user’s activity in terms of location and time spent, which is critical as a mobility tracking tool. This information can be categorised into six community spaces: retail and recreation; parks; groceries and pharmacies; workplaces; transit stations; and residential areas. (Sulyok and Walker, 2020) The mobility assessment can be done by understanding the relative changes in visitors at each community space, which is calculated by comparing the percentage change in the number of visitors at each of these community spaces in comparison to the baseline period.

Due diligence has been taken in the comparison of individuals on each particular day of the week, given the fact that the daily routine of individuals is different on weekdays and weekends. The period between January 3, 2020 and February 6, 2020, spread over 5 weeks, with the median value, is the baseline period. It also provides baseline days, which indicate the normal value on each particular day of the week and allow the study of mobility changes in subsequent lockdown phases.

The main intent of this mobility assessment is to compare the percentage change in mobility from the pre-pandemic baseline, across the six spatial dimensions, in the state of Tamil Nadu between the phases of lockdown. The eight time windows have mobility distribution for each community space. We compare the mobility distribution between time periods and initially check for normality assumptions by adopting the Shapiro–Wilk test. Most distributions are non-normal, and a statistically significant difference is found using the Wilcoxon test. Finally, the measure of effect size, Cliff’s delta, is computed as it is robust when the distributions are non-Gaussian.

## Results

### Descriptive statistical analysis

The following paper has two periods of interest: the pre-pandemic period from January 1, 2010 to March 22, 2020, and the pandemic period from March 23, 2020 to December 31, 2021. The descriptive statistics of the daily count of missing person cases registered in Tamil Nadu such as mean, median, mode, and standard deviation are 32, 32, 18, 14 and 55, 67, 63, and 21 for the pre-pandemic and pandemic periods, respectively (see Table 1). It would not be prudent to straightaway infer that the pandemic period witnessed a massive jump in the recorded level of missing person cases. It is essential to consider the trend, seasonality, and holiday effects of the time-series data before coming to any conclusion regarding the impact of the pandemic. A general increasing trend of cases related to missing persons can be seen across the years in Fig. 2. The counterfactual prediction shows the daily count of cases registered if lockdown had not been imposed. The actual or observed daily count of cases is available as well. It is reiterated that the difference between the two is the magnitude of the causal impact of lockdown.

**Table 1:** Descriptive statistics: central tendencies for all crimes in both pre-pandemic and pandemic periods.

Type of crime	Mean	Median	Mode	Std. dev.
Pre-pandemic period (1 January 2010 to 22 March 2020)				
Unidentified dead bodies	12.075	12	13	5.858
Missing person	32.440	32	18	14.417
Pandemic Period (23 March 2020 to 31 December 2021)				
Unidentified dead bodies	16.985	17	18	4.963
Missing person	54.891	57	63	20.885

### Results of mobility trends in Tamil Nadu and Chennai

Given the methodology adopted, mobility trends in Tamil Nadu in general and Chennai in particular have been analysed. The results are discussed below.

The imposition of lockdowns by the government aimed to contain the infection and prevent its spread to newer areas. These orders impacted the mobility levels of individuals across various activity zones, as well as the movement of goods and services and vehicles. The Google Community Mobility database was used for both Tamil Nadu as a whole and also Chennai, the largest metropolitan city in the state, separately, for the relevant time periods of waves one and two to assess mobility patterns. With the stay-at-home lockdown orders, a massive spike in terms of percentage change in relation to the baseline value in the mobility levels in the residential neighbourhoods can be observed. However, significant declines in percentage change in mobility in comparison to the baseline values in all other spheres of activities like grocery and pharmacy, retail and recreation, transit stations, workplaces, and parks can also be observed. Table 2 shows that the reduction levels seem to be higher in the complete and partial lockdowns of the first wave than in the second wave.

**Table 2:** Descriptive statistics of the changes in mobility (in %) from baseline during various lockdown phases in both waves in Tamil Nadu in the six spheres of activity.

Period/zone		Retail and recreation	Grocery and pharmacy	Parks	Transit stations	Workplaces	Residential
CL 2020	Mean	−80.4	−52.21	−39.38	−62.03	−64.33	32.10
	Std	11.85	17.71	8.79	9.30	11.70	5.72
	Max	−26	21	−15	−24	−29	39
PL 2020	Mean	−70.21	−16	−40.82	−41.56	−39.21	22.77
	Std	8.87	14.17	4.7	8.91	14.31	5.49
	Max	−54	5	−32	−26	−6	36
Post-L 2020	Mean	−39.65	−0.57	−36.8	−22.7	−24.13	13.3
	Std	4.8	7.8	3.68	3.7	11.7	2.8
	Max	−34	18	−29	−16	3	19
PL–One 2021	Mean	−47	−4.73	−34.04	−33.08	−36.19	19.42
	Std	11.57	16.15	9.17	10.88	9.94	3.79
	Max	−25	27	−15	−13	−11	26
CL 2021	Mean	−60.42	−22.15	−40.03	−47.67	−44.61	23.55
	Std	5.96	8.87	6.39	5.27	8.56	2.8
	Max	−4	1	−25	−35	−25	28
PL–Two 2021	Mean	−35.07	9.31	−19.66	−23.97	−27.48	13.93
	Std	7.93	10.46	5.27	6.52	5.33	−15
	Max	−20	28	−9	−12	−15	19
	Mean	−6.8	45.6	9.16	4.2	−8.23	8.7
	Std	6.4	9.2	5.7	5.6	12.12	2.3
	Max	8	68	23	23	16	15

The mobility trends were primarily similar in the comparative analysis between Chennai and Tamil Nadu. The differential impact in Chennai on mobility was more pronounced than in Tamil Nadu. In both waves, in all phases and across all spatial domains, Chennai witnessed a more significant percentage change in mobility. See Table 3.

**Table 3:** Descriptive statistics of the changes in mobility (in %) from baseline during various lockdown phases in both waves in Chennai in the six spheres of activity.

Period/zone		Retail and recreation	Grocery and pharmacy	Parks	Transit stations	Workplaces	Residential
CL 2020	Mean	−88.87	−61.94	−88.48	−85.48	−78.6	36.38
	Std	8.52	19.38	8.52	5.8	9.48	6.3
	Max	−46	18	−56	−58	−48	45
PL 2020	Mean	−80.5	−39.3	−92.2	−75.5	−62.07	30.07
	Std	23.3	9.8	2.98	4.65	11.88	5.39
	Max	−69	−25	−84	−68	−32	41
Post-L 2020	Mean	−51.7	−16.7	−68.7	−52.7	−45.9	17.01
	Std	5.87	9.8	5.55	4.9	9.64	3.16
	Max	−46	1	−55	−44	−24	23
PL–One 2021	Mean	−46.3	−1.69	−48.69	−40.42	−40.3	16.19
	Std	15.5	26.9	15.49	14.12	14.89	5.04
	Max	−29	29	−25	−26	−7	27
CL 2021	Mean	−79.3	−43	−76.18	−72.7	−66.8	31.03
	Std	8.24	24.3	6.4	11.6	17.3	7.55
	Max	−55	23	−60	−40	−11	40
PL–Two 2021	Mean	−54.82	−4.3	−55.9	−49.4	−49	19.37
	Std	12.09	14.6	9.76	12.14	11.1	5.24
	Max	−33	19	−37	−26	−22	29
Post-L 2021	Mean	−23.83	24.23	−24.3	−16.6	−28	9.83
	Std	7.4	11.52	7.47	5.4	12.07	2.9
	Max	−15	43	−16	−9	−3	20

**Fig. 6:**
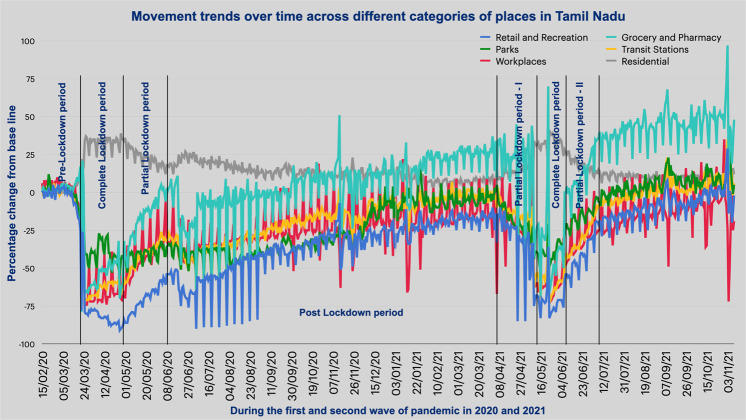
Percentage changes in mobility from baseline—six spatial domains—Tamil Nadu—various phases—both waves. *x*-axis—dates various lockdown phases—Tamil Nadu—both waves in 2020 and 2021. *y*-axis: percentage change in mobility for six spatial domains from baseline. The vertical bars space out the different lockdown phases such as CL—complete lockdown, PL—partial lockdown and Post-L—post lockdown. Source: Google Community Mobility Reports.

The mobility levels are assessed across the six spatial domains in eight windows of the lockdown period. The research is based on the lockdown phases; the missing person cases registered during these time intervals are analysed when mobility is significantly affected. The inferences are drawn in terms of the factors affecting the registration of missing person cases and unidentified dead bodies (UIDB). Besides, Cliff’s delta is utilised as a measure of the effect size (E.S.) to interpret the differences in mobility in these comparative periods (Paramasivan et al., 2022). The results were as anticipated; there was a significant spike in the percentage change from the baseline value concerning residential neighbourhoods due to stay-at-home orders. However, in Tamil Nadu, there was a steep decline in the percentage change in mobility from the baseline of all other spheres of activities like retail and recreation, grocery and pharmacy, parks, transit stations, and workplaces (see Fig. 6). Notably, the quantum of such reduction was greater for complete lockdown and partial lockdown in the first wave than in the second wave, as shown in Table 4.

**Table 4:** Effect size for various group comparisons among various phases of lockdown during first and second waves of COVID-19 in the state of Tamil Nadu and city of Chennai.

Cliff’s delta effect size		PL-2020 vs. CL-2020	PLs-2021 vs. CL-2021	CL-2020 vs. CL-2021	PL-2020 vs. PLs-2021
Tamil Nadu	Retail and recreation	0.548, large	0.53, large	−0.52, large	−0.73, large
	Grocery and pharmacy	0.47, medium	0.51, large	−0.35, medium	−0.85, large
	Parks	0.06, negligible	0.49, large	−0.23, small	−0,84, large
	Transit stations	0.47, medium	0.47, large	−0.25, small	−0.35, medium
	Workplaces	0.56, large	0.54, large	−0.29, small	−0.35, medium
	Residential	−0.51, large	−0.5, large	0.14, small	0.44, medium
Chennai	Retail and recreation	0.53, large	0.527, Large	−0.59, large	−0.69, Large
	Grocery and pharmacy	0.44, medium	−0.51, large	−0.35, medium	−0.79, large
	Parks	0.13, negligible	0.53, large	−0.67, large	−0.88, large
	Transit stations	0.49, large	0.50, large	−0.61, large	−0.76, large
	Workplaces	0.55, large	0.54, large	−0.43, large	−0.52, large
	Residential	−0.45, medium	−0.53, large	0.38, medium	0.65, large

### Results of counterfactual analysis of time series

**Fig. 7:**
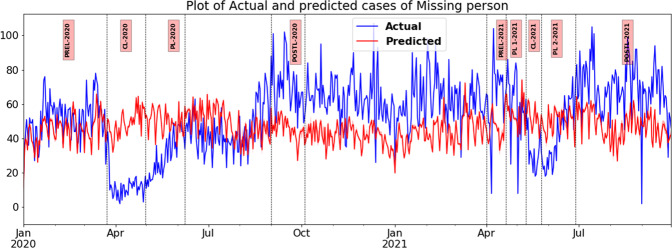
Plot of actual and predicted number of missing person cases using an auto-regressive neural network. *x*-axis—timeline of various lockdown phases in both waves in Tamil Nadu. *y*-axis—daily count of missing person cases registered (actual and predicted). The vertical bars space out the different lockdown phases such as CL—complete lockdown, PL—partial lockdown and Post-L—post lockdown.

**Fig. 8:**
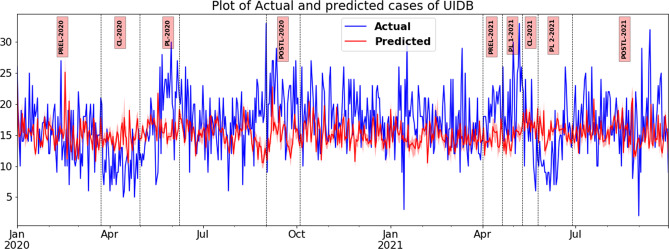
Plot of actual and predicted number of unidentified dead body (UIDB) cases using an auto-regressive neural network. *x*-axis—timeline of various lockdown phases in both waves in Tamil Nadu. *y*-axis—daily count of unidentified dead body (UIDB) cases registered (actual and predicted). The vertical bars space out the different lockdown phases such as CL—complete lockdown, PL—partial lockdown and Post-L—post lockdown.

In accordance with the study’s assumption regarding the effect of mobility on the reporting of cases involving missing persons in Tamil Nadu, there was a substantial decline in registered cases when compared to the counterfactual during the complete lockdowns of both waves, with a more significant decline in CL-2020 (−74% and E.S.-0.981) and a moderate decline in CL-2021 (−36% and E.S. 0.74). The number of registered cases of unidentified dead bodies (UIDB) followed a similar pattern, albeit with a lesser magnitude of decline (see Fig. 7). When compared with the counterfactual, the recorded instances of UIDB decline of −26% E.S.-0.68 in CL 2020 and a minor dip of −6.12% ES-0.22 in CL 2021. In the phases of PLs during wave one in 2020 and wave two in 2021, there was an increase in mobility from CL to PL. However, the declining trend of missing persons and UIDB cases continued in waves one and two of PLs. Though smaller than what was observed during CLs, the decline in registration remained significant, with a reported large effect size in wave one (−0.84 in PL-2020) and a small one in wave two (−0.20 in PL-Two-2021) (see Fig. 8). In wave two, when curbs were removed, the number of registered cases of missing persons increased by 40% (ES + 0.78) and the number of UIDB cases increased by 18% (ES + 0.39). Chennai City underwent the same analysis again. Chennai experienced a similar pattern to what was observed in Tamil Nadu, in the missing person reports during all phases of both pandemic waves. However, the magnitudes of decline and increase during CLs and PLs were greater in Chennai City than in Tamil Nadu. (please refer to Tables 5 and 6).

**Table 5:** Actual and predicted weekly counts of violent crimes during various phases of lockdowns in waves one and two in Tamil Nadu in 2020–21.

Crime/period	UIDB	Missing person (not traced)
CL-2020		
Actual mean	10.71	12.74
Predicted mean	14.63	48.74
Confidence interval	11.11, 19.02	32.42, 64.08
Percentage change	−26.77	−73.87
Cliff’s delta	−0.68	−0.981
PL-2020		
Actual mean	17.95	30
Predicted mean	15.94	49.72
Confidence interval	13.76, 20.4	35.66, 61.04
Percentage change	12.59	−39.66
Cliff’s delta	0.204	−0.839
Post-L-2020		
Actual mean	20.42	70.26
Predicted mean	15.03	45.39
Confidence interval	10.97, 20.86	27.74, 56.92
Percentage change	26.41	35.4
Cliff’s delta	0.666	0.931
PL-One 2021		
Actual mean	20.7	60.25
Predicted mean	14.54	55.2
Confidence interval	11.59,17.43	32.88,59.76
Percentage change	42.32	9.14
Cliff’s delta	0.65	0.33
CL-2021		
Actual mean	15.8	33.67
Predicted mean	16.83	52.03
Confidence interval	11.59,17.43	32.88,59.76
Percentage change	−6.12	−35.29
Cliff’s delta	−0.218	−0.742
PL-Two 2021		
Actual mean	12.71	42.74
Predicted mean	15.62	50.07
Confidence interval	12.6,19.34	35.56,59.58
Percentage change	−18.6	−14.63
Cliff’s delta	−0.513	−0.264
Post-L-2021		
Actual mean	17.53	63.53
Predicted mean	14.87	45.39
Confidence interval	11.85, 19.71	34.54, 57.99
Percentage change	17.93	39.98
Cliff’s delta	0.393	0.784

**Table 6:** Actual and predicted weekly counts of violent crimes during various phases of lockdowns in waves one and two in chennai in 2020–21.

Crime/period	UIDB	Missing person (not traced)
CL-2020		
Actual mean	73.5	16.17
Predicted mean	121.30	29.60
Percentage change	−39.41	−45.39
Cliff’s delta	−1.00	−0.94
PL-2020		
Actual mean	125.8	29.7
Predicted mean	127.9	29.1
Percentage change	−1.6	2.0
Cliff’s delta	0.11	0.00
Post-L-2020		
Actual mean	133.6	81.0
Predicted mean	124.2	23.3
Percentage change	7.6	247.0
Cliff’s delta	0.346	1
PL-One 2021		
Actual mean	244.7	63.0
Predicted mean	133.0	71.8
Percentage change	83.9	−12.2
Cliff’s delta	1.00	−0.78
CL-2021		
Actual mean	200.7	44.0
Predicted mean	134.8	75.1
Percentage change	48.8	−41.4
Cliff’s delta	0.33	−1.00
PL-Two 2021		
Actual mean	149.3	46.0
Predicted mean	130.4	80.0
Percentage change	14.5	−42.5
Cliff’s delta	0.11	−1.00
Post-L-2021		
Actual mean	127	81
Predicted mean	112.6	34.31
Percentage change	12.7	136
Cliff’s delta	0.5	1

## Discussion

**Fig. 9:**
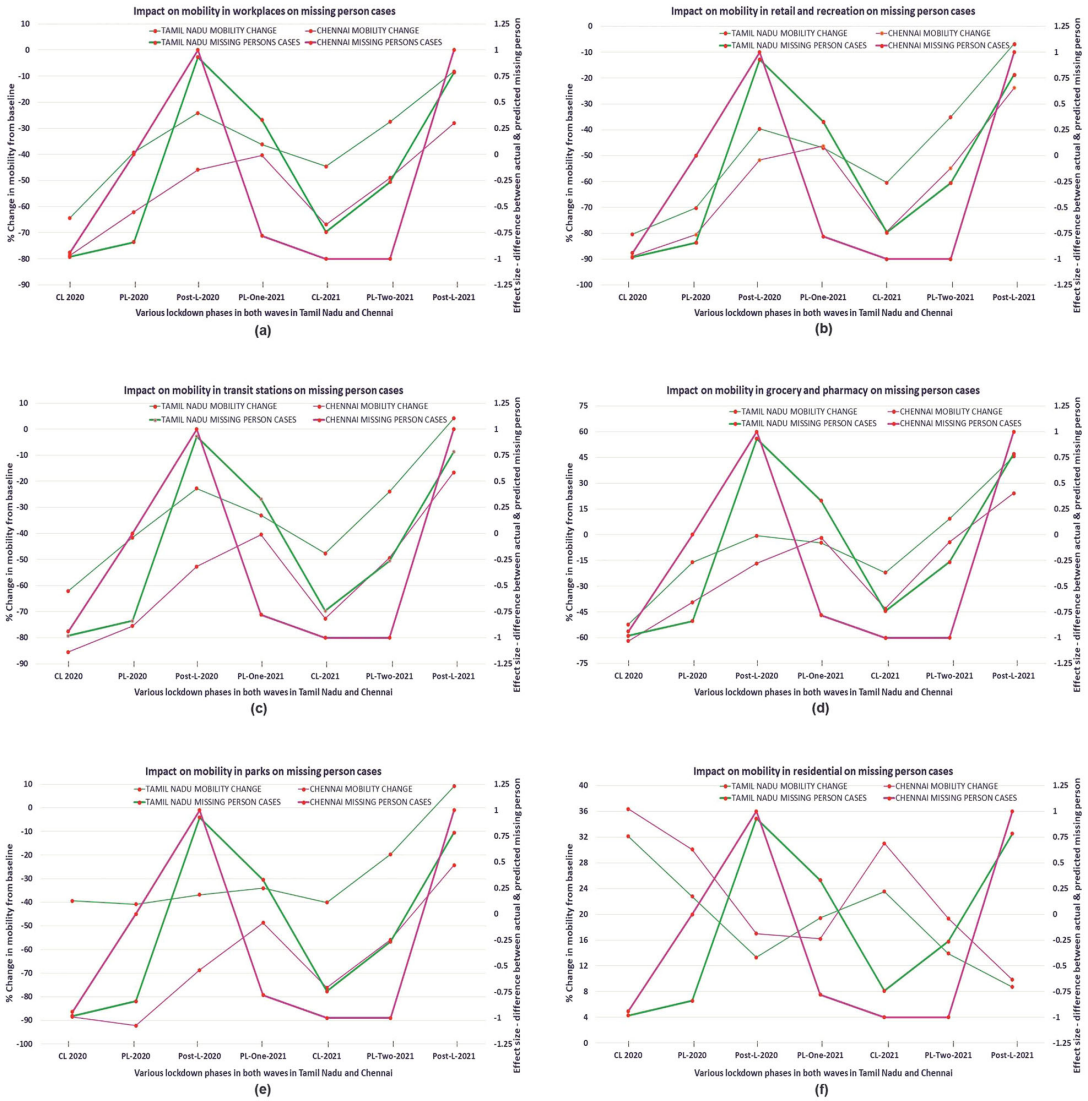
Impact of mobility on registered cases of missing person during pandemic. Relationship between mean percentage change in mobility from baseline and difference between actual and predicted of missing person cases (effect size-Cliffs Delta)—six spatial domains—Tamil Nadu and Chennai. Panel (left): **a**, **c**, **e** Workplace, Transit stations and parks (Mobility vs. effect size (difference between actual cases registered and predicted, expressed as Cliffs delta). Panel (right): **b**, **d**, **f** Retail and recreation, grocery and pharmacy, residential (Mobility vs. effect size (difference between actual cases registered and predicted, expressed as Cliffs delta). *x*-axis—discrete intervals showing various lockdown phases (CL is complete Lockdown, PL is partial lockdown, and Post-L is post-lockdown). *y*-axis (left)—shows the mean percentage change in mobility from baseline in different community spaces. *y*-axis (right)—shows the Cliffs delta, a measure of effect size (difference between the actual and predicted cases of registered missing person cases in Tamil Nadu and Chennai) across all spaces and in various lockdown phases.

The results above clearly show that there was a drastic reduction in registered cases of missing persons during periods of decreased mobility. This trend is clearly discerned during the transition phases from complete lockdown to partial lockdown in both waves. When all curbs or restrictions on mobility were removed, especially during the second wave in the post-lockdown phase, there was a spike in the number of cases reported. This could be attributed to improved reporting channels. The study thoroughly examines changes in the six spatial domains to note the impact on situational factors for both Tamil Nadu and Chennai City (see Fig. 9).

The mobility trends and changes in registered missing person cases were analysed to check for urban-rural consistency. In the five spatial domains, namely, “retail and recreation”, “grocery and pharmacy”, “workplace”, “transit stations”, and “parks”, and across all the lockdown periods, there was a greater percentage reduction in mobility with baseline in Chennai than in Tamil Nadu. Within the spatial domains, a more pronounced differential impact was noticed in “workplace”, “transit stations”, and “parks” than in “retail and recreation” and “grocery and pharmacy”. In the same way, as expected, there was a bigger rise in mobility in “residential” areas of Chennai than in Tamil Nadu.

The reporting channels of missing persons are primarily dependent on the availability of transport, restrictions enforced, and the accessibility of police officers. This directly relates to mobility in the six spatial domains discussed above. The six graphs in the figure indicate that increasing trends in mobility are strongly associated with escalated registered cases of missing persons compared with the counterfactual. The contra-trend of decreasing mobility in residential areas, meaning when fewer individuals are at home, with surging cases of missing persons, is along the same lines. The pandemic-induced lockdown may have impacted only mobility directly, but its reverberations were felt in almost all the situational factors that determine the registration of missing person cases. The crucial situational factor that influences reporting and registration of missing person cases is the ability of complainants to access the relief centres, including the police. Faster, quicker, and easier modes of transportation and their availability to the complainant and the police are likely to result in a higher number of registered cases.

Situational factors came to the fore during stricter phases of lockdowns, during post-lockdown phases, and in the period between the two waves, where more enabling channels of reporting resulted in a significantly higher number of cases registered.

As stated above, upon investigation, the cases of missing persons may turn out to be homicide, abduction, kidnapping, and human trafficking. While these were not crimes at the time of registration, they must be included in the analysis because the restrictive orders caused large-scale dislocation of people, particularly in the context of the labour class, which included guest workers from northern Indian states who had come in search of work in Tamil Nadu. Besides the closure of many institutions, a new culture of working from home has also contributed to the dislocation of individuals from their earlier places of living. In the cases registered as missing persons, there was a drastic decline of −73.87 and −35.29% during CL-2020 and CL-2021 and, similarly, a sharp escalation of 35.40 and 39.98% during PL-2020 and PL-2021. During PL-2020, PL-One-2021, and PL-Two-2021, the changes in percentages were −39.66, +9.14, and −14.63, respectively. It is noticed that there is a decline in the recorded levels of missing persons during CLs and PLs in both the waves, whereas there was a very sharp escalation during the post-lockdown phases of both waves. It is a general practise that the kith and kin do not immediately report instances of missing persons as they usually hope and wait for the missing person to return. This delayed lodging of complaints has always been present, even in the pre-pandemic period. The increased level of reporting and registration of cases during the post-lockdown periods of both waves points to the inability of complainants to reach out to agencies to locate the missing persons during both periods of complete as well as partial lockdowns. This delayed lodging of complaints further highlights the inadequacy of existing registration channels.

### Missing persons and UIDB

In fact, the cases of missing persons, where the kith and kin are involved in reporting and following up on the resolution of these cases, present an important study not just to understand them in isolation, but also to trace the correlation they bear with cases of unidentified dead bodies, which remain unresolved for ages, given that there are no immediate kith and kin to report to. In fact, as policing experiences show, the cases of missing persons and unidentified dead bodies are related in several ways. A dead body found in a place whose identity is not known is registered as a case of an unidentified dead body in the jurisdictional police station. This case may have provenance to a case of a missing person registered at some other police station. Furthermore, the origin, cause, or reason for a missing person’s case may not always be the same as that of an unidentified dead body. However, for a given place, such as a district or state, over some time, the case trends for the missing person and the unidentified dead body seem to be very similar. The case of a missing person could be viewed as the first and foremost instance where the police take cognisance of a problem. This could manifest and result in a case of homicide, suicide, or accident, and the deceased’s identity has not been established at the other police station. The resultant case is registered as a case of an unidentified dead body.

### Policy implications

The problems encountered in resolving cases related to missing persons are numerous. As suggested by this study, the issues surrounding the resolution of missing persons are not just related to the filing of the cases, but also to issues of general access to reporting. The panic-driven and lockdown-induced migration during the pandemic led to the forced dislocation of many individuals (Mishra, 2021). The trauma associated with the loss of lives during the pandemic, the loss of livelihood due to job losses, and the enhanced isolation due to forced lockdowns could all be possible reasons for “missing persons” cases. These may not be relevant during non-pandemic times, or indeed could be, but given that the registration of cases and ability to investigate is smoother in non-pandemic times, the emphasis on the investigative aspect is given more impetus rather than the pre-investigative aspects. The pandemic provides a natural setting to examine the pre-investigative reasons and hence the factors that rarely come into focus during regular times. Hence, this study allows for a closer examination of the importance of mobility in various spatial scenarios in the registration of cases, which can thereby help solve missing persons cases better in the future.

As has already been enunciated, finding ways to resolve these issues has to be one of the police force’s most important tasks at hand. It is in line with this thought that this study has been conducted. Conclusions have been provided to generate a separate body of literature to support the analysis of the factors affecting the registration of cases of missing persons, factors which pertain not just to pandemic times but also to non-pandemic times, when they would otherwise not be noticed but are nonetheless equally relevant in missing person cases.

With the possibility of exceptional circumstances, it is essential to be well prepared, given that everyday lives cannot be affected for long. As the world witnessed, lockdowns cannot be imposed indefinitely, nor can normal life be hindered for too long. Further, in the context of this paper, relaxation in individuals’ movements saw an upsurge in the filing of missing cases, which goes on to emphasise that during the panic-stricken pandemic times, with regulations in place, individuals are deprived of accessing agencies, such as the police, and seeking appropriate remedies. This situation requires amendment, and citizens should have access to justice at all times, where there are pandemics or any other circumstances that affect mobility. Hence, this paper recommends policy reorientation and dynamic changes at multiple levels, including coordination between state and central agencies, proactive participation of non-profit organisations, support mechanisms for victims, etc., to ensure that during times of hardship, the mechanism is in place for individuals to have adequate access to criminal justice agencies.

“Missing persons” is a sensitive issue for family members, given the protracted agony that they may face in the absence of any knowledge about the whereabouts and situation of their loved ones. (Holmes, 2016) Hence, police personnel need to be sensitised as to how to handle these cases, trained to follow Standard Operating Procedures and handle the issue swiftly, given that most of the studies highlight the importance of acting within the first 48 hours or so following the FIR (Briones and Chhabra, 2022; Pierzchala et al., 2021), in order to maximise the chances of tracing the missing person. Often the focus of the missing individual is “body centred” and less on forensic investigative principles. A higher reporting requirement, alongside ensuring prompt action, also facilitates clear roles and responsibilities of all stakeholders involved, through an integrated, multidisciplinary, and multi-agency approach (Salado Puerto et al., 2021).

Technological intervention is inevitable as it helps to identify and analyse challenges that could prevent registration, such as mobility, as demonstrated by this paper. This then results in delays in the process of filing and investigation. Mobility can thus also be enhanced by augmenting technology and engaging communities at various levels. As a result, in addition to the intervention of Crime Criminal Tracking Network Systems (CCTNS), a centralised pool of data on the missing people and a repeated release of missing persons list may be needed. A profiling tool can be created and stored as a database. This will serve as a ready reckoner in adverse situations. Registers have to be produced with a standardised operating procedure and a proper understanding of the spatial features of the police division topography. This must include awareness of age and gender diversity; socio-economic profiles of the individuals in these locations, such as the awareness of the diversity of the population in terms of school and college students and marginal classes; awareness of the dark alleys of the cities and crowded places; and search techniques linking the probable locations based on age, gender, and occupational diversity of the missing persons. Many reports from around the world (Parr and Fyfe, 2013) have emphasised this kind of evidence-based policing. A case in point is the success that the Australian Police met with, in utilising the media to assist in the location and recovery of absent people (Siddiqui and Wayland, 2022).

Missing persons, either voluntary or involuntary, can ultimately be linked to the psycho-social-cultural elements of society, as with any abnormality or crime. Repeated missing is a case wherein the person missing runs away on more than one occasion, given the pressures at home, or mental health issues. In order to stop missing cases of this nature from happening again, it is important to raise awareness about mental health within the community.

In the short run, socio-economic resources play a vital role in individuals going missing. Hence, keeping crime in check and focusing policing on specific potentially problematic sections of the society, such as the youth, unemployed, and illness-prone individuals, is crucial. This will help the police keep ears to the ground, as will special awareness camps on the problems and rights of individuals, to ensure the police has close access to individuals, which in turn can keep the “missing person phenomenon” both voluntary and involuntary, in check. Moreover, governance-related interventions are integral to solving issues of policing. In particular, governance reforms in terms of enhancing police resources, both human and infrastructural, are integral to ensure that adequate focus is placed on all forms of crime, including missing persons.

## Conclusions

The pandemic witnessed one of the largest migrations of individuals in India after the partition of India in 1947. Owing to the cessation of public transport during certain critical phases of the lockdowns, individuals were displaced and dislocated, contributing to higher missing person cases. In absolute numbers, the daily count of the mean number of missing person cases rose by 61%, that is, from 32 in the pre-pandemic period (Jan 1, 2010 to March 22, 2020) to 55 in the pandemic years (March 23, 2020 to December 31, 2021). The daily count of the mode number of cases during the corresponding periods was 18 and 63, which also highlights the huge upsurge of cases.

The case of missing persons has been a testimony to the significance of mobility, thus highlighting the indispensable role of registration of crimes and setting the law in motion. The study clearly suggests that during CL, there was a decline of 74% and 36% of missing person cases in the actual case when compared with the counterfactual, during the first and second waves, respectively. There was a corresponding escalation of 35% and 40% in the post-lockdown phases. The only exception was that when there were no restrictions during the two years, the actual number of registered cases was consistently higher than predicted in all other periods. While we acknowledge that the pandemic has adversely impacted the reporting channels of all crimes, it is more acute in the cases of missing persons as locating them gets more challenging with the passage of time on account of delayed reporting. Hence, there is a dire need to provide better physical access for these complainants, provide adequate relief centres, and involve all stakeholders to jointly work towards a quick redressal of grievances by promptly locating and tracing the missing persons.

## Data Availability

The data that support the study’s findings are available from the State Crime Records Bureau of the Tamil Nadu Police Department. The author does not have permission to share the data for public use. However, upon a reasonable request, the data can be made available for research purposes after the researchers get consent from the Tamil Nadu State Crime Records Bureau.
